# Intravitreal Injections in Arc Sterile Setting: Safety Profile after More Than 10,000 Treatments

**DOI:** 10.1155/2020/3680406

**Published:** 2020-04-15

**Authors:** Claudio Furino, Maria Oliva Grassi, Vito Bini, Annalisa Nacucchi, Francesco Boscia, Michele Reibaldi, Nicola Recchimurzo, Giovanni Alessio

**Affiliations:** ^1^Eye Clinic, Azienda Ospedaliero-Universitaria Policlinico, University of Bari, Bari, Italy; ^2^Eye Clinic, Department of Ophthalmology, University of Sassari, Sassari, Italy; ^3^Eye Clinic, Department of Ophthalmology, University of Catania, Catania, Italy

## Abstract

**Purpose:**

To report the occurrence of endophthalmitis and other complications after intravitreal injections (IVIs) in the Arc Sterile setting.

**Methods:**

A retrospective study that enrolled all patients who underwent IVIs between November 2017 and March 2019, collecting data about the patient's gender and age, type of injected drug, diagnosis, other ocular pathologies, physician and possible occurrence of endophthalmitis, or other complications.

**Results:**

Ten thousand and eighty-three IVIs were performed during the study period, involving 2014 eyes of 1,670 patients with an average age of 71.37 ± 11.63 years. The injected drugs included ranibizumab (54.6%), aflibercept (38.0%), dexamethasone (6.7%), pegaptanib (0.3%), bevacizumab (0.4%), and ocriplasmin (0.01%). The diagnosis included neovascular age-related macular degeneration (859), myopic choroidal neovascularization (154), diabetic macular edema (576), retinal vein occlusion (203), and miscellaneus diagnosis (222). No cases of endophthalmitis were recorded. One hundred and sixty-nine cases of ocular hypertension were detected, while the most frequent complication was subconjunctival hemorrhage, identified after 1,180 IVIs. The residents performed over 80% of IVIs, but there was no statistically significant difference in incidence of complications between the residents group and consultants group.

**Conclusions:**

Arc Sterile seems to be a safe setting in which IVIs can be carried out, regarding infective risk, and it is easy to set up compared to operation theatre and useful to improve intravitreal injections governance.

## 1. Introduction

Intravitreal injection (IVI) is the most common performed ophthalmic procedure. It is currently used in the treatment of neovascular age-related macular degeneration, myopic neovascularization, and in macular edema due to retinal vein occlusion, diabetes, or other pathologies [1]. Usually, intravitreal injections are performed in two different settings, operating room setting or office-based setting [2].

**Figure 1:**
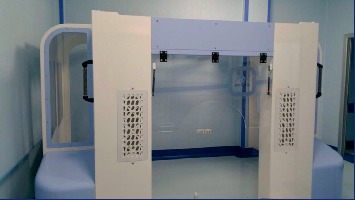
Arc Sterile in place: the structure has a frontal aperture for the entry of patients; on either side, two columns provide filtration of the air. Two sterile horizontal laminar flows are driven to the center of the cabin and cross themselves on the patient's head.

Recently, a new controlled ambient surgical cabin to perform IVIs has received the CE Mark for distribution in Europe, named as Arc Sterile (Arc Sterile, Spain) [3–5] (Figure 1).

This portable system can be easily wheeled and quickly set up, turning any room into an operating theatre. Arc Sterile is an ISO 5 class device; it means that it guarantees the limit of 3,520 uncontrolled particles in one m^3^ of air reducing the number of microorganisms in it (bacteria, fungi, and viruses) and preventing the sedimentation of microorganisms inside the wounds. This is possible because the laminar flow sweeps over the surgical area with clean air without turbulence. In this way, it moves the contaminated air away from the operation field [6].

One of the most potentially devastating complications secondary to IVI remains infectious endophtalmitis, with a rate of 0.056% [7], but also some other ocular adverse events can occur. The most frequent are intraocular sterile inflammation, rhegmatogenous retinal detachment, traumatic cataract, intraocular pressure elevation, and ocular vitreous hemorrhage [8]. The rate of endophthalmitis and other complications of IVIs performed in theatre setting compared to the community setting is not known. In addition, the results of the studies are extremely various [2, 9–11].

The aim of the study was to evaluate complications of IVIs performed in Arc Sterile setting in a context of a high volume tertiary Italian center.

## 2. Materials and Methods

From November 2017, the Arc Sterile has been introduced in our hospital (Eye Clinic, Azienda Ospedaliero-Universitaria Policlinico, University of Bari, Italy) and has become the gold standard setting where to perform IVs; it was located within an ambulatory surgery center. Our model (MB30) was 3 meters wide. A specific electronic database was created that included baseline patients' characteristics, surgical details, and follow-up (scheduled one day and one month after the injection).

Patients were asked to take two-day preoperatory therapy consisting of antibiotic eye drops (ofloxacin 3 times a day) before IVI. Topical anaesthesia was achieved using benoxinate eye drops; povidone-iodine 5% was initially used to sterilize the ocular surface and conjunctival sac. Patients were positioned inside the Arc Sterile cabin, in order to secure the eye in the sterile area generated by laminated horizontal flows. A sterile eyelid speculum was placed having the eyelid disinfected with a povidone-iodine 5% solution before. The physician carried out the injection as per protocol [12]. The treating physicians wore sterile surgical clothing and used a sterile scrub brush soaked with povidone-iodine before wearing sterile gloves. Moreover, surgical masks, hairnets, sterile drapes, and overshoes were used in order to minimize every risk of infection.

Drugs injected were 0.5 mg/0.05 ml ranibizumab (Lucentis®, Genentech Inc., South San Francisco, CA, USA and Novartis AG, Basel, Switzerland), 0.3 mg pegaptanib (Macugen®; Eyetech Pharmaceuticals Inc., FL, USA and Pfizer Inc., New York, NY, USA), 2 mg/0.05 ml aflibercept (EYLEA®; Regeneron Pharmaceutical Inc., Tarrytown, NY, USA and Bayer, Basel, Switzerland), 1.25 mg/0.05 ml bevacizumab (Avastin®; Genentech USA, Inc.), 0.125 ml/0.1 ml ocriplasmin (Jetrea®; ThromboGenics, Iselin, New Jersey, USA), and intravitreous dexamethasone implants (Ozurdex®, Allergan Inc., Irvine, CA, USA).

Bevacizumab for injection was drawn from the vial in a clean room located in the Service of Pharmacy; pegaptanib and aflibercept were prepared by the treating physician immediately before the injection; ocriplasmin was diluted and prepared by the physician; all of the liquid drug vial content was withdrawn through a sterile filter and a 19-gauge (0.912 mm) needle attached to a 1 mL sterile Luer lock syringe. The filter needle was replaced with a sterile 30-gauge (0.255 mm) needle for the intravitreal injection. Ranibizumab was supplied in prefilled syringes, but the same sterile 30-gauge needle was needed. Dexamethasone implant required a specific applicator with a 22-gauge (0.644 mm) needle. The ocriplasmin injection was preceded by anterior chamber paracentesis, in order to prevent sustained intraocular pressure elevation after the procedure.

The procedure ended with the instillation of topical antibiotic and povidone-iodine 5% drops. After the procedure, patients were discharged home under medical therapy. All IVIs were performed by 5 senior surgeons and by 10 residents under the supervision of the attached consultant. The potential impact of the residents' learning curve on complications was also monitored. Any intraoperative adverse events, such as conjunctival lacerations, lens injury, choroidal, or vitreal hemorrhage, were recorded.

Postoperative medications accounted of topical ofloxacin 3 times a day for 5 days. Patients were asked to attend control visit the day after the procedure and at one month. The control visit consists of slit-lamp biomicroscopy, applanation tonometry, and when necessary, fundus examination by indirect ophthalmoscopy. During the follow-up, postoperative adverse events and complications associated with IVIs were evaluated, like endophthalmitis, intraocular inflammation, rhegmatogenous retinal detachment, intraocular pressure (IOP) elevation (we considered an IOP cut-off level of 23 mm Hg for ocular hypertension diagnosis), and ocular haemorrhage. The most devastating complication was the infectious endophthalmitis; the diagnosis based on the purulent inflammation of aqueous and vitreous. According to the Endophthamitis Vitrectomy Study (EVS) [13], we suspected this complication when the patient complained pain, red eye, and blurring. Usually, common signs of endophthalmitis were conjunctival congestion, corneal edema, anterior chamber cells and fibrin, hypopyon, vitreous inflammation, retinitis, and retinal periphlebitis. In any case of clinical suspicion, a vitreous tap or even a core-vitrectomy was required to study microbiological characteristics of intraocular fluid.

### 2.1. Statistical Analysis

Data are presented as mean and standard deviation (SD) on median and interquartile range (IQR). Categorical variables were reported as the percentage. Intergroup comparisons were performed with the *χ*^2^ test (categorical variables). All analyses and data modelling were performed using R-project (R Core Team 2013, Vienna, Austria; http://www.R-project.org).

## 3. Results

From November 2017 to March 2019, 10,083 intravitreous injections were performed in 1,670 patients with an average age of 71.37 ± 11.63 years. Females were 793 (47.5%). Three hundred and torty-four (20.6%) patients received intravitreal injections in both eyes, so 2.014 eyes were enrolled in the study. One thousand two hundred and twenty-nine (61%) eyes were pseudophakic, 785 (39%) were phakic, and 83 (4.1%) eyes were vitrectomized.

The diagnosis included neovascular age-related macular degeneration (42.7%), myopic choroidal neovascularization (7.6%), diabetic macular edema (28.6%), retinal vein occlusion (10.1%), and miscellaneus diagnosis (11.0%) (Table 1).

Data are presented with mean and standard deviation, median and interquartile range, or number and %. IVIs: intravitreal injections; nAMD: neovascular age-related macular degeneration; mCNV: myopic choroidal neovascularization; DME: diabetic macular edema; RVO: retinal vein occlusion; MIX: miscellaneus.

Type of drug injected is shown in Table 2.

Injections were performed by 5 consultants (group A: *n* = 2.017) and 10 residents (Group B: *n* = 8.066). There were no major intraoperative complications. All patients attended the day-1 visit. No cases of endophthalmitis were recorded; subconjunctival hemorrhage was identified in 1,180 eyes (11.7%). One-month follow-up was 97% completed, and during this second visit, no cases of endophthalmitis were recorded, but 169 cases of ocular hypertension were detected (1.7%). There was no statistically significant difference in incidence of overall complications (ocular hypertension and subconjunctival hemorrhage) between the consultants and resident injections groups (264 (13.1%) vs. 1.085 (13.5%) group A vs. B, respectively, *p*=0.72) (Table 3).

## 4. Discussion

The main finding of this study is that the IVI performed in the Arc Sterile context is safe and effective. To our knowledge, this is the first report on the use of Arc Sterile in a tertiary setting. We observed an enhanced incidence of adverse events in the trainee group in relation to the learning curve; however, that did not result in serious infective complications.

Intravitreal injection is a surgical procedure easy to perform. The side and adverse effects are rare, but the high volume of performed on international scale makes the IVI complications a severe issue for the ophthalmologists [14]. The most dramatic complication is endophthalmitis that potentially can lead to complete functional impairment or even to evisceration [15]. According to Italian Ophthalmological Society (SOI), intravitreal injections should be provided by an ophthalmic surgeon and must be carried out in an operating theatre, in order to prevent infective risks [16]. This is a suboptimal cost-effective option because it requires the simultaneous use of many operating rooms, high-volume staffing, and prolon0ged patients turn-over that may result in misuse of economic resources and ultimately in increasing the patients waiting time.

Arc Sterile is an innovative strategy to perform intravitreal injection. It reduces the number of suspended particles in the air by means of double filtration, laminated flow, air renewal, and positive pressure [6]. Therefore, the surgical field may be considered aseptic and satisfies the requirements of ISO (International Organization for Standardization) 5 quality level air classification, mandatory for an operating room [17]. Moreover, Arc Sterile is a mobile cabin that can be positioned in any room, with no restrictions or specific regulatory requirements. In our study, we used a floor area corresponding to 10% of our operating suite.

Data collected in the first 16 months from inauguration of the new surgical setting are very encouraging: among 10,083 intravitreal injections performed, with an average of 33 procedures a day, no case of infective ocular complications was recorded. This result was lower than the incidence rate reported in the meta-analysis carried out by Fileta et al., which identified 197 cases of endophthalmitis among 350,535 IVI of anti-VEGF (0,056%) [7]. Xu et al. recently showed a reduced incidence of endophthalmitis after IVI performed in an outpatient clinic setting (40 cases among 258,357 IVI), without topical antibiotic eyedrops after the injection; this retrospective study considered only anti-VEGF agents (bevacizumab, ranibizumab, and aflibercept), injected using a 30-gauge needle [18].

Mishra et al. reported the rate of endophthalmitis after 20,566 IVI of anti-VEGF using a 30-gauge needle and triamcinolone acetonide (TA) which required a 26-gauge (0.405 mm) needle. In their series, the procedures were performed in an operating room with both pre- and postinjection antibiotic prophylaxis, and they recorded an overall incidence of endophthalmitis of 0.131%. The adverse event occurred only in patients treated with TA and bevacizumab, especially if bevacizumab injections were drawn from a vial, instead of no cases of infection out of prefilled ranibizumab syringe. Therefore, the authors supported the theory that the smaller needle and the prefilled syringes correlated with a lower risk for infective complications [19].

Compared to these previous large studies, our sample was relatively small but included also 8% of IVI of Ozurdex® that required an applicator with a 22-gauge needle. Despite larger size of needle used for dexamethasone, we did not observe endophthalmitis.

The risk of management mistakes was theoretically very likely because the whole staff (physicians, optometrists, nurses, and porters) for the first time worked in a team, using a new instrument in new rooms. The variable which had the likelihood to induce complications in this new operating setting was the amount of ophthalmic surgeons with different skills (consultants and ophthalmologist in training): nine different physicians carried out the injections; so, during the time of study, each of them was starting to gain confidence at the new instrument. Lastly, most of IVIs were performed by residents, who had less experience than consultants, even if under the surveillance of a skilled surgeon. Despite all these possible risk factors, the incidence rate in our experience with Arc Sterile was 0%.

Arc Sterile setting also decongested the operating theatre, which can be reserved for more complex surgeries. Moreover, this new organization allowed us to perform intravitreal injection every day, in order to reduce waiting time between diagnosis and the first drug administration and to respect the timing of medications.

This study has the following limitations: is a single cohort observational study with no case-control; no cost-effectiveness; and no patients' satisfaction analysis were carried out.

In conclusion, although the study period was limited, IVIs in Arc Sterile setting is safe and easy to set up. To confirm our data, a larger sample is required.

## Figures and Tables

**Table 1:** Baseline characteristics from 1670 patients.

*n* (%)	*n* eyes = 2,014
	*n* IVIs = 10,083
Gender distribution	
Male	877 (52.5)
Female	793 (47.5)
Age (years, median [SD])	71.37 ± 11.63

Laterality	
Unilateral	1326 (79.4)
Bilateral	344 (20.6)

Lens status	
Phakic	785 (39.0)
Pseudophakic	1229 (61.0)

Diagnosis	
nAMD	859 (42.7)
mCNV	154 (7.6)
vDME	576 (28.6)
RVO	203 (10.1)
MIX	221 (11.0)

**Table 2:** Distribution of IVIs based on drugs injected.

Drugs *n* (%)	Number of injections
Ranibizumab	5504 (54.6)
Aflibercept	3832 (38.0)
Dexamethasone	676 (6.7)
Pegaptanib	30 (0.3)
Bevacizumab	40 (0.4)
Ocriplasmin	1 (0.01)
Tot.	10083 (100)

Data are expressed as number and percentage.

**Table 3:** Postoperative complications evaluated in consultants and residents subgroups.

*n* (%)	Total	Consultants	Residents	*p*
Injections	10083 (100)	2017 (20.0)	8066 (80.0)	

Complications	1349 (13.4)	264 (13.1)	1085 (13.5)	0.72
Endophthalmitis	0 (0)	00	0	—
Ocular hypertension	169 (1.7)	34 (1.7)	135 (1.7)	0.85
Subconjunctival hemorrhage	1180 (11.7)	230 (11.4)	950 (11.8)	0.71

## Data Availability

The data used to support the findings of this study are available from the corresponding author upon request.

## References

[1] Merani R., Hunyor A. P. (2015). Endophthalmitis following intravitreal anti-vascular endothelial growth factor (VEGF) injection: a comprehensive review. *International Journal of Retina and Vitreous*.

[2] Tabandeh H., Boscia F., Sborgia A. (2014). Endophthalmitis associated with intravitreal injections. *Retina*.

[3] MA F. (2015). Arc Sterile: a cost-effective luxury. *Revista de Enfermeria*.

[4] Ruão M., Andreu-Fenoll M., Dolz-Marco R., Gallego-Pinazo R. (2017). Safety of bilateral same-day intravitreal injections of anti-vascular endothelial growth factor agents. *Clinical Ophthalmology*.

[5] http://www.Arc.Sterile.com/

[6] http://www.imex.es/Arc Sterile/

[7] Fileta J. B., Scott I. U., Flynn H. W. (2014). Meta-analysis of infectious endophthalmitis after intravitreal injection of anti-vascular endothelial growth factor Agents. *Ophthalmic Surgery, Lasers and Imaging Retina*.

[8] Ghasemi Falavarjani K., Nguyen Q. D. (2013). Adverse events and complications associated with intravitreal injection of anti-VEGF agents: a review of literature. *Eye*.

[9] Abell R. G., Kerr N. M., Allen P., Vote B. J. (2012). Intravitreal injections: is there benefit for a theatre setting?. *British Journal of Ophthalmology*.

[10] Brynskov T., Kemp H., Sørensen T. L. (2014). No cases of endophthalmitis after 20,293 intravitreal injections in an operating room setting. *Retina*.

[11] Casparis H., Wolfensberger T. J., Becker M. (2014). Incidence of presumed endophthalmitis after intravitreal injection performed in the operating room. *Retina*.

[12] Frenkel R. E. P., Haji S. A., La M. (2010). A protocol for the retina surgeon&rsquo;s safe initial intravitreal injections. *Clinical Ophthalmology*.

[13] Endophthalmitis Vitrectomy Study Group (1995). Results of the endophthalmitis vitrectomy study. *Archives of Ophthalmology*.

[14] Lai T. Y. Y., Liu S., Das S., Lam D. S. C. (2015). Intravitreal injection-technique and safety. *Asia-Pacific Journal of Ophthalmology*.

[15] Al-Rashaed S., Alsulaiman S., Alrushood A., Almasaud J., Arevalo J. (2016). Incidence of endophthalmitis after intravitreal Anti-vascular endothelial growth factor: experience in Saudi Arabia. *Middle East African Journal of Ophthalmology*.

[16] Società Oftalmologica Italiana (SOI) (2017). Linee di indirizzo iniezione di farmaci per via intravitreale (iv). *Quarto Aggiornamento Marzo*.

[17] Istituto superiore per la prevenzione e la sicurezza del lavoro (2009). Dipartimento igiene del lavoro. Linee guida sugli standard di sicurezza e di igiene del lavoro nel reparto operatorio. https://www.inail.it/cs/internet/docs/linee-guida-igiene-reparto-operatorio.pdf?section=attivita.

[18] Xu K., Chin K., Bennett R. (2018). Endophthalmitis after intravitreal injection of vascular endothelial growth factor inhibitors: management and visual outcomes. *Ophthalmology*.

[19] Mishra C., Lalitha P., Rameshkumar G. (2018). Incidence of endophthalmitis after intravitreal injections: risk factors. *microbiology profile, and clinical outcomes. Ocular Immunology and Inflammation*.

